# Our Empire State Edition

**Published:** 1898-05

**Authors:** 


### EDITORIAL.

### OUR EMPIRE STATE EDITION.

WE present with this number our good wishes to the veterinarians of the Empire State. Her devotees of the profession have ever been among those who have given generous support to veterinary journalism, and her record in college, association, education, and publication work has won for her the warm appreciation of the entire profession. To her eight hundred devotees the JOURNAL extends its best wishes and greetings.

